# Revisiting the role of attention in the “weapon focus effect”: Do weapons draw gaze away from the perpetrator under naturalistic viewing conditions?

**DOI:** 10.3758/s13414-022-02643-8

**Published:** 2023-02-01

**Authors:** Hannes M. Körner, Franz Faul, Antje Nuthmann

**Affiliations:** Institute of Psychology, Kiel University, Olshausenstr. 62, 24118 Kiel, Germany

**Keywords:** Weapon focus effect, Attention, Eye movements, Eyewitness memory, Dynamic scenes

## Abstract

The presence of a weapon in a scene has been found to attract observers’ attention and to impair their memory of the person holding the weapon. Here, we examined the role of attention in this weapon focus effect (WFE) under different viewing conditions. German participants viewed stimuli in which a man committed a robbery while holding a gun or a cell phone. The stimuli were based on material used in a recent U.S. study reporting large memory effects. Recording eye movements allowed us to test whether observers’ attention in the gun condition shifted away from the perpetrator towards the gun, compared with the phone condition. When using videos (Experiment 1), weapon presence did not appear to modulate the viewing time for the perpetrator, whereas the evidence concerning the critical object remained inconclusive. When using slide shows (Experiment 2), the gun attracted more gaze than the phone, replicating previous research. However, the attentional shift towards the weapon did not come at a cost of viewing time on the perpetrator. In both experiments, observers focused their attention predominantly on the depicted people and much less on the gun or phone. The presence of a weapon did not cause participants to recall fewer details about the perpetrator’s appearance in either experiment. This null effect was replicated in an online study using the original videos and testing more participants. The results seem at odds with the attention-shift explanation of the WFE. Moreover, the results indicate that the WFE is not a universal phenomenon.

Weapons have been shown to attract more attention and gaze than other objects (Biggs et al., 2013; E. F. Loftus et al., 1987). Moreover, the presence of a weapon in a scene can impair an observer’s memory for relevant aspects of the environment, in particular for the person holding the weapon (e.g., E. F. Loftus et al., 1987; Mitchell et al., 1998; Pickel, 1998, 1999, 2009). This phenomenon is known as the weapon focus effect (WFE). While the memory effects of weapons are well established (see Fawcett et al., 2013, and Kocab & Sporer, 2016, for meta-analyses), very few studies have explicitly addressed the attention component of the WFE. The main goal of the present research was to test whether attentional effects of weapons found for slide shows (E. F. Loftus et al., 1987) and non-narrative static images (Biggs et al., 2013) generalize to more naturalistic viewing conditions (i.e., videos). Memory for the perpetrator’s appearance was also assessed.

Currently, there exist two main theoretical explanations for the WFE: the arousal/threat hypothesis and the unusual item hypothesis. The arousal/threat hypothesis attributes the phenomenon to the threatening nature of weapons (e.g., Hulse & Memon, 2006; Maass & Köhnken, 1989). Based on Easterbrook’s (1959) “cue utilization hypothesis,” the arousal induced by a weapon is assumed to cause a narrowing of attention, leading witnesses to focus on central details (i.e., the weapon itself) at the cost of attention to peripheral ones (i.e., other aspects of the perpetrator and the scene). In contrast, the unusual item hypothesis highlights the unusualness of weapons in most situations (e.g., Mitchell et al., 1998; Pickel, 1998). Objects that are unexpected in the given context have been shown to attract more attention and eye fixations than context-congruent objects (e.g., G. R. Loftus & Mackworth, 1978). Weapons are unusual in most contexts. Therefore, according to the unusual item hypothesis, weapons often form a special case of incongruent objects (E. F. Loftus et al., 1987).

A central assumption underlying both theoretical approaches is that the weapon draws attention to itself, which leads to poorer encoding of other aspects of the scene. Eye tracking provides researchers with an excellent tool for assessing how overt attention is allocated in the scene (e.g., Foulsham, 2015; Williams & Castelhano, 2019). In particular, fixation locations reveal *where* participants attend to in a scene, whereas fixation durations indicate *how long* attention and gaze remain at a specific location (Henderson, 2003). Two eye-tracking studies on the WFE provided evidence for the assumption that weapons attract attention and gaze (Biggs et al., 2013; E. F. Loftus et al., 1987). E. F. Loftus et al. (1987) recorded the eye movements of participants who were asked to view a slide show of a man taking money from a cashier. Memory for the man was worse when he carried a gun than when he held a check. Importantly, eye-movement data revealed that the weapon was fixated more often and for longer durations than the neutral object.

In the study by Biggs et al. (2013), participants were presented with several unrelated, non-narrative images (i.e., images involving different people and settings), each depicting a person holding either a weapon or a neutral object. On trials including a weapon, participants spent more time fixating what was being held, thereby replicating E. F. Loftus et al. (1987). In line with current theories on the WFE, Biggs et al. (2013) found that this attentional focus on the weapon came at a cost in viewing time on the face of the person. Biggs et al. (2013) further manipulated participants’ action-specific potential during stimulus presentation by either arming participants with a gun or giving them a metal cylinder to hold. Holding a gun in a readily usable position led to a general bias towards faces, which did not interact with the observed attentional WFE.

Other studies that examined the attentional component of the WFE used either very short exposure durations (Flowe et al., 2013; Harada et al., 2015) or did not record participants’ eye movements (Erickson et al., 2014; Hope & Wright, 2007). Erickson et al. (2014) used a self-report measure to assess observers’ attentional allocation. They found that participants who saw a target person carrying a weapon or an unusual object compared with a neutral object indicated to have paid more attention to the object and less attention to the face of the person. If this self-report is accurate, these results suggest that an attentional shift due to weapon presence is consciously accessible.

In contrast to previous research, Scrivner et al. (2019) found indirect evidence that weapons do not always attract attention. In their study, the authors did not specifically examine the effects of weapons, but instead tracked subjects’ eye movements while they viewed images of violent vs. non-violent interactions. Viewing time on the objects held by the depicted people did not differ between violent and non-violent scenes. Instead, when viewing violent scenes, participants attended more to locations where the people touched each other and less to faces. There are some aspects of this study that limit the generalization to the WFE: Given the focus of the study, some of the objects in violent interactions were not weapons, and not all weapons present in the scenes were held by the interacting people. Moreover, a few of the non-violent scenes included (holstered) weapons. Perhaps more importantly, some of the stimuli depicted police officers carrying weapons, and it has been shown that the presence of a gun has no effect on memory when it is carried by a police officer (Pickel, 1999).

In studies on the WFE, the scenarios in which the weapon is presented, the control of intervening variables, and the way in which the effect is measured, vary considerably (Kocab & Sporer, 2016). In part, this variability can be attributed to the conflicting goals arising from the applied perspective, which demands a highly realistic setting, and the basic research perspective, which places more emphasis on stimulus control. Using videos of staged criminal scenarios presents a good compromise in this regard, as videos allow researchers to depict realistic scenarios and control the stimulus on basic dimensions (e.g., Mitchell et al., 1998; Pickel, 2009; Pickel & Sneyd, 2018).

A recent meta-analysis suggests that the mode of presentation (slides vs. video/live) does not modulate the memory-related WFE (Kocab & Sporer, 2016). The effect of presentation mode on the attentional component of the WFE is less clear, because previous eye-tracking studies used slide shows (E. F. Loftus et al., 1987) or non-narrative static images only (Biggs et al., 2013). Thus, despite the complexities that arise when analyzing eye movements observers make when viewing dynamic scenes, it is important to test whether findings obtained for static scene images generalize to moving images (i.e., videos).

Research on eye-movement control during naturalistic scene viewing has shown that gaze behavior depends on both the mode of stimulus presentation and the viewing task (Dorr et al., 2010; Smith & Mital, 2013). Dorr et al. (2010) compared participants’ eye movements while they watched 20-s long videos of real-world scenes containing no cuts and only minimal camera movement as opposed to random static images or stop-motion sequences (i.e., slide shows of a selection of still images taken from the respective video). When viewing stop-motion sequences or random static images, most participants focused their attention on the center of the screen at the start of the presentation of a new image or slide. This center bias (e.g., Buswell, 1935) is known to be particularly prominent shortly after stimulus onset (Tatler, 2007). Accordingly, for the first second following a new slide, observers’ gaze behavior was more similar in the stop-motion sequence compared with the same point in the video condition (Dorr et al., 2010). After this initial phase, however, coherence dropped below the level observed in the video condition. Thus, the inter-observer coherence showed different time courses in videos and stop-motion sequences: stop-motion sequences led to an alternating rise and fall of coherence, whereas coherence in the video condition maintained a more stable level.

It is currently unclear whether and how the effects of weapon presence on attention change over time. In previous studies in which attention shifts towards weapons were found, the exposure duration for the critical object (i.e., the weapon or the neutral control) was relatively short (6 s in E. F. Loftus et al., 1987, and 5 s per image in Biggs et al., 2013). It is quite possible that the presence of a weapon affects the allocation of attention within the first few seconds only.

Culture may constitute another factor moderating the WFE. Most published studies on the WFE were conducted in the U.S. Gun ownership is a constitutional right in the U.S., whereas gun laws are much more restrictive in other regions, including most European countries. It seems plausible that varying “gun cultures” could affect the processing of gun-related events. The comparatively few studies that examined the effects of weapons on memory outside of the U.S. produced mixed results. Some studies found a classic WFE (Hope & Wright, 2007; Kim et al., 2014; Maass & Köhnken, 1989; Saunders, 2009), but only Hope and Wright (2007) included a gun as a weapon. In contrast, several recent European studies found that the WFE occurred only under certain conditions (Harvey & Sekulla, 2021; Mansour et al., 2019) or not at all (Harvey et al., 2020; Nyman et al., 2020). All of these more recent studies have in common that even reversed effects were found for some measures or experimental conditions.

A more specific finding potentially affected by culture is a moderating influence of the perpetrator’s race on the strength of the WFE. Pickel and Sneyd (2018) reported a weaker memory-related WFE for a Black than for a White perpetrator. The authors attributed this difference to racial stereotypes linking weapons to Black men, thus rendering them less unusual in this context. However, racial stereotypes are less universal than, for example, gender stereotypes and may differ significantly across nations (see Fiske, 2017, for a review). It is therefore unclear how well the findings by Pickel and Sneyd (2018), obtained from a Midwestern U.S. population, generalize to other cultures. Recent results by Frenken et al. (2022) suggest that similar racial stereotypes exist in Germany. Frenken et al. (2022) used a computer-based first-person shooter task (Correll et al., 2002) for which several U.S. studies have found that participants are quicker to “shoot” armed Black actors compared with White ones and slower to indicate a “don’t shoot” response for unarmed Black actors (Mekawi & Bresin, 2015). Frenken et al. (2022) found that this racial bias generalizes to German participants.

The goal of the current multi-experiment study was to investigate some of the open research questions outlined above. The purpose of Experiment 1 was to examine the effects of weapon presence on attention using dynamic stimuli and longer exposure durations. The stimuli were derived from the video footage used in Experiment 1 by Pickel and Sneyd (2018), in which a significant memory-related WFE for details of the perpetrator’s appearance was found. Eye tracking was used to explicitly test the key assumption that the memory-related WFE is caused by an impaired encoding of the perpetrator’s appearance due to a shift of the observer’s attention away from the perpetrator and towards the weapon. Moreover, conducting our study in Germany allowed us to test whether cultural differences regarding the role of weapons in society and stereotypes affect the strength of the WFE. Finding such differences would mean that cultural specificities need to be taken into consideration when estimating the effects of weapon presence on eyewitness testimony.

To preview the results: Contrary to predictions by current theories and results from previous eye-tracking studies on the WFE (Biggs et al., 2013; E. F. Loftus et al., 1987), the weapon did not draw significantly more gaze than a neutral object, and weapon presence had no effect on viewing time for the perpetrator. Weapon presence also did not affect observers’ memory performance, which means that we could not replicate the memory-related WFE.

To further investigate these unexpected results, we conducted two additional experiments. In Experiment 2, we used stop-motion versions of the videos to replicate the methodology of E. F. Loftus et al. (1987). Our eye-tracking results were compatible with their observations, but we still failed to find a memory-related WFE. In Experiment 1, we used modified versions of the videos used by Pickel and Sneyd (2018). To test whether these modifications could explain the differences regarding the memory effects, we conducted a third experiment in which the original videos were presented online. Again, no memory effect was found.

## Experiment 1

In our main Experiment 1, German participants watched videos derived from the material of Experiment 1 in Pickel and Sneyd (2018) while their eye movements were tracked. The videos showed a man, who was either Black or White, committing a robbery. The perpetrator was holding either a weapon or a neutral (i.e., non-threatening and context-congruent) control object. After watching the video, participants were asked to fill out a questionnaire regarding the appearance of the perpetrator. Additionally, we used a self-report measure of attention to check (a) whether a potential shift of attention is consciously accessible (as the results of Erickson et al., 2014, suggest) and (b) how well self-reported eye movements match the actual gaze behavior.

If the common assumptions about the attentional effects of weapons are correct, then viewing time on the weapon should be longer compared with the neutral object, which should in turn come at a cost in viewing time on the perpetrator. As a consequence of this attentional shift, the results from the memory questionnaire should reveal a reduced accuracy for details of the perpetrator’s appearance, as observed in many previous studies, including Pickel and Sneyd (2018).

Moreover, Pickel and Sneyd (2018) found that the WFE was weaker for a Black perpetrator than for a White perpetrator, which they explained with racial stereotypes. If similar stereotypes exist in Germany, the WFE should be reduced for the Black perpetrator. Under the assumption that an attentional shift towards the weapon is indeed the key mechanism underlying the memory-related WFE, the time spent looking at the weapon versus the neutral object should be more similar for the Black perpetrator than for the White one. Alternatively, if racial stereotypes linking weapons to Black men are less pronounced in Germany, no effect of perpetrator race on either attention or memory may be observed.

### Methods

#### Design and participants

The experiment had a 2 (object type: gun and cell phone) × 2 (perpetrator race: Black and White) between-subjects design. We conducted an a priori power analysis to determine the sample size required to detect large effects (η^2^ ≥ .14) with analyses of variance. In our study, most variables known to moderate the memory-related WFE (e.g., the exposure duration to the critical object) were within the ranges that produced the strongest effects in previous studies (Fawcett et al., 2013). Moreover, Pickel and Sneyd (2018), whose stimulus material we adapted, found large effects of weapon presence on memory. The power analysis yielded a required sample size of *N* = 68 to achieve a power of at least 1 − β = .90 with a significance level of α = .05. Accordingly, the sample consisted of 68 participants (52 women, 16 men) between 19 and 43 years of age (*M* = 23.9 years, *SD* = 4.9). Of these, 65 self-identified as White, whereas three reported being non-White. None self-identified as Black. Most participants were undergraduate psychology students. Subjects received either monetary compensation or course credit for their participation. The experiment was approved by the Central Ethics Committee of Kiel University, and participants gave informed consent accordingly.

#### Stimuli

The videos were adapted from Experiment 1 in Pickel and Sneyd (2018). The original videos were about 90 seconds long and showed a man robbing two other men while carrying an object. The perpetrator was either Black or White and was holding either a handgun or a cell phone. Thus, there were four different versions of the video. The videos subtended 24.1° horizontally and 18.2° vertically. Since they were filmed in a 4:3 format, but presented on a 16:9 monitor, the left and right edges of the screen were set to black (4.1° each).

The perpetrator was visible during two separate scenes of the video. In the first scene, he walked down the corridor of an office building while looking around. No other people were visible during this part of the video. In the second scene, the perpetrator entered a room where the two victims were working, one of them Black, the other White. He took their wallets and tablets before leaving. The perpetrator was visible for approximately 6 s in the first scene and for approximately 26 s in the second scene. The critical object was always with him and constantly visible to the observer. Each of the scenes consisted of a single medium long shot.

Compared with the study by Pickel and Sneyd (2018), two changes were made to the videos. The protagonists in the videos spoke English. Therefore, and firstly, we chose to present the videos to our German subjects without sound, because we wanted to eliminate any potential effects of varying degrees of comprehension or increased cognitive load due to processing a foreign language (Roussel et al., 2017). Importantly, numerous studies have found a WFE when presenting stimuli without sound (e.g., Mitchell et al., 1998; Pickel, 1998, 1999), suggesting that omitting the soundtrack should not matter.

Secondly, we chose to exclude a section of the video showing a close-up on the critical object. In the original version, when the perpetrator was shown for the first time, the shot started with a close-up on the object he carried. This focus was maintained for around 3 s before the camera slowly zoomed out to the medium long shot. In our experiment, the scene began with the zoomed out shot. For one, the close-up constitutes an unnatural element, thereby interfering with our goal to study the allocation of attention under naturalistic viewing conditions. Moreover, as no other objects of interest were present during the close-up, it is highly likely that participants would fixate and process the critical object during this part of the video. This could in turn reduce the need to further process the object later on, when both the perpetrator and the object become visible. Thus, the close-up potentially reduces the need for participants to choose between the critical object and the perpetrator when allocating their attention.

#### Apparatus

A 24 in. monitor with a resolution of 1920 × 1080 pixels and a refresh rate of 144 Hz was used for stimulus presentation. Eye movements were recorded using an SR Research EyeLink 1000 Desktop Mount. The 2000 Hz camera upgrade allowed for binocular tracking with 1000 Hz for each eye. A chin and forehead rest was used to minimize participants’ head movements. The viewing distance was 93 cm.

#### Procedure

Participants were instructed to pay close attention to the content of the video, without disclosing that their memory for certain aspects of the video would be tested afterwards. Before stimulus presentation, a nine-point calibration and validation of the eye tracker was performed. Moreover, the following fixation check was implemented: A fixation cross was presented at the center of the screen for 500 ms. The video was displayed only if the participant’s gaze deviated less than 0.6° from the cross for at least 200 ms. Immediately after participants had watched the video, they filled out the questionnaire regarding the appearance of the perpetrator.

The memory questionnaire was a German version of the questionnaire used by Pickel and Sneyd (2018). The questionnaire consisted of questions regarding the appearance of the perpetrator, both in a single choice format (e.g., “Was the perpetrator wearing a jacket?” with the response categories “yes” and “no”) and in an open format (e.g., “What color was the jacket of the perpetrator?”). To increase the number of responses per subject, we added a set of questions about the perpetrator’s backpack.

The awareness and endorsement of the stereotype that Black men are more likely to commit crimes involving weapons was assessed using rating scales. Participants were asked whether, according to (a) existing stereotypes (stereotype awareness) and (b) their personal beliefs (stereotype endorsement), a Black or a White man was more likely to commit a crime using a weapon.

We added a new question to assess participants’ introspection regarding their eye movements: The task was to indicate what percentage of time they thought they had looked at each of five predefined regions during the sequences in which the perpetrator was visible. These regions were the perpetrator, the object he held, the Black victim, the White victim, and any region outside of these categories. Note that this method differs from the unidimensional scale employed by Erickson et al. (2014), who asked participants to indicate whether they had paid more attention to the face of the target person or the object she was holding. We chose to assess the self-reported viewing time for different regions in order to be able to directly and in detail compare objectively measured and subjectively reported viewing times.

#### Data analysis

The data were analyzed using SR Research Data Viewer, Python, and R (see Appendix 1 for software details). Eye movements were not analyzed for the entire videos. Instead, analysis was limited to the sequences where the perpetrator was visible (i.e., the hallway scene and the robbery scene). The empirically determined quality of the eye-tracking data was high (see Appendix 2). For a given participant, the data from the eye with the lower average error during the eye-tracker validation were analyzed (cf. Hooge et al., 2019).

#### Creation of dynamic regions of interest

**Fig. 1:**
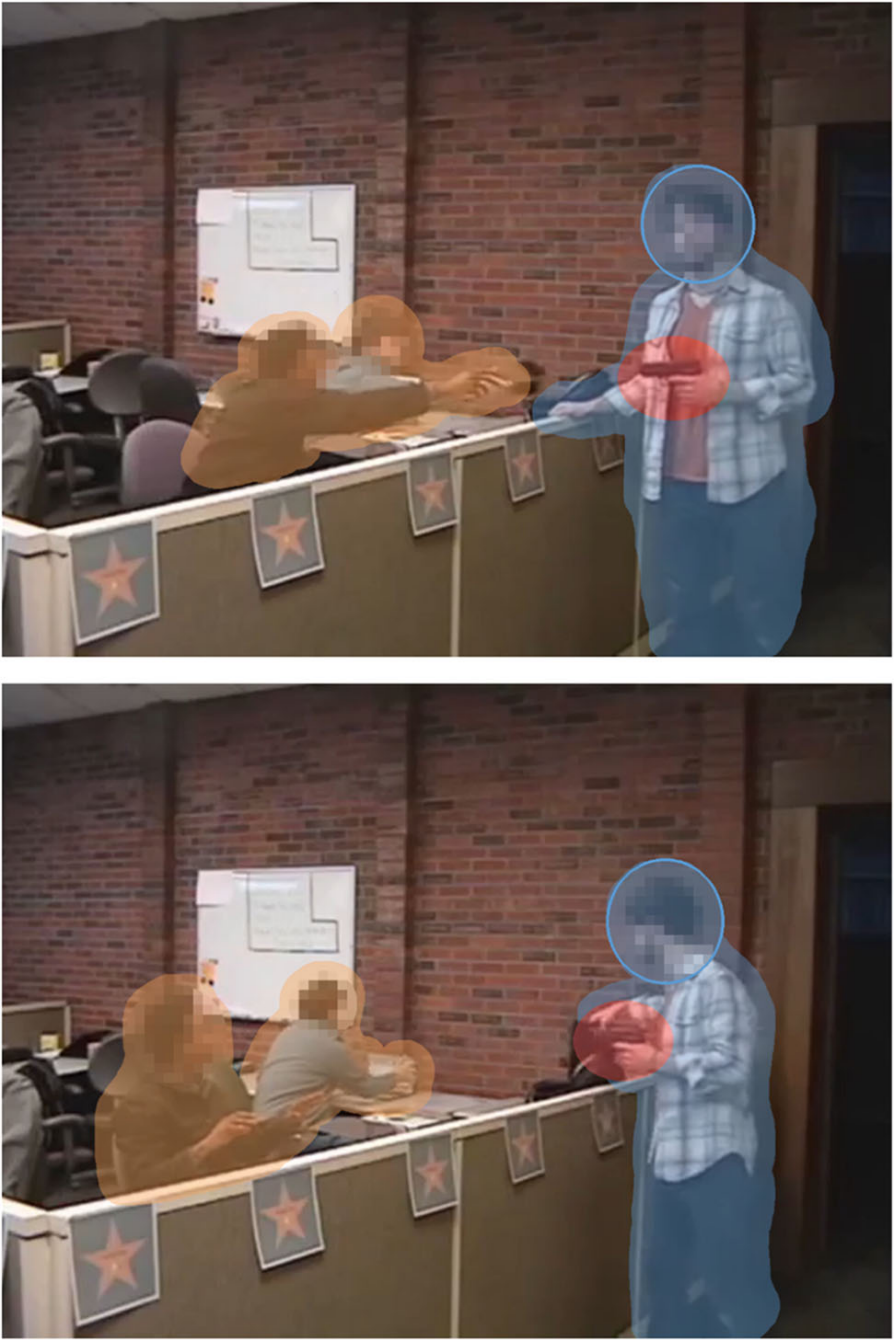
Example frames for the gun and the White perpetrator with superimposed regions of interest. Top panel: The victims hand over their wallets. Bottom panel: The perpetrator takes a tablet. Transparent overlays mark the regions of interest for the perpetrator (blue), the critical object (red), and the victims (orange). The blue ellipses mark the borders of the region of interest for the perpetrator’s head. Faces were pixelated for privacy protection; note that this was not the case in the experimental stimuli. (Color figure online)

To analyze the allocation of attention and gaze, dynamic regions of interest (ROIs) were defined around the perpetrator, the victims, and the critical object (see Fig. 1). The ROIs for the perpetrator and each of the victims were generated automatically using the neural network module *PointRend* (Kirillov et al., 2020) with a ResNet-101 backbone. For the critical object, an elliptical ROI was created manually in SR Research Data Viewer. Given that human faces are generally known to attract attention and the eyes (e.g., Cerf et al., 2009), a separate elliptical ROI was created for the head of the perpetrator (cf. Biggs et al., 2013). For our main analyses, viewing times on the perpetrator were calculated by adding the viewing times on his head and his body. Similarly, viewing times were added up for the two victim ROIs, essentially treating the two victim ROIs as a single ROI covering both victims. To account for inaccuracies in the eye-tracking hardware, a margin of approximately 0.5° was added to each ROI (cf. Orquin et al., 2016).

When generating ROIs for the critical object and the perpetrator’s head in Data Viewer, instances were created manually for some of the video frames. The software then automatically interpolated the ROI coordinates with an interval of 30 ms. If necessary, further instances were added until the ROI closely matched the actual movement of the target.

Some of the created ROIs overlapped. Specifically, the ROI for the critical object always overlapped with the ROI for the perpetrator. Naturally, the ROI for the perpetrator’s head also overlapped with the ROI for the entire perpetrator. The ROI for the critical object did not overlap with the victim ROI. There was also virtually no overlap between the perpetrator ROIs and the victim ROI, except for short overlaps when the victims handed over the items and when the perpetrator left.

Due to these overlaps, gaze samples could fall into more than one ROI. In these cases, samples were assigned to only one of the ROIs based on a predefined hierarchy. The ROI of the critical object took priority over the ROIs of the perpetrator. Within the perpetrator ROIs, samples were assigned primarily to the head and then to the automatically generated ROI covering the entire perpetrator. The ROI of the victims had the lowest priority.

#### Smooth pursuit check

When viewing dynamic scenes, observers sometimes follow moving objects steadily with their gaze. These eye movements are known as smooth pursuit (see Goettker & Gegenfurtner, 2021, for a review). The EyeLink event parser cannot detect smooth pursuit eye movements, but instead classifies them as fixations (Smith & Mital, 2013). However, intrafixational displacements of the gaze position are expected to be larger in the presence of smooth pursuit. To check for the presence of smooth pursuit in each event classified as a fixation, we determined the Euclidian distances between the gaze coordinates of the last sample of the previous saccade and the first sample of the next saccade (cf. Hutson et al., 2017; Nuthmann & Canas-Bajo, 2022). We then compared the distances with those observed in Experiment 2 where slide shows of single frames from the videos were used. There were larger displacements for the videos (*M* = 0.65°, *SD* = 0.11) than for the slide shows (*M* = 0.36°, *SD* = 0.09), *t*(122) = 15.48, *p* < .001, *d* = 2.79 (one-tailed test). These results indicate that the eye-movement data obtained with the videos included some smooth pursuit epochs.

For hypothesis testing, we calculated the viewing times for different ROIs rather than analyzing the durations of individual fixations. Therefore, we chose not to exclude potential smooth pursuit movements. However, the presence of smooth pursuit segments was taken into account when assigning the eye movement data to ROIs: Rather than using the mean gaze coordinates for oculomotor events classified as fixations (the default in Data Viewer), ROI matching was based on the individual gaze samples.

#### Memory scores

The answer key for correct responses regarding the appearance of the perpetrator was adapted from Pickel and Sneyd (2018). Participants’ responses were scored independently by two raters who were unaware of the underlying hypotheses. Ratings were aggregated across both raters. For each participant, the number of correctly and incorrectly reported details was determined. When evaluating the open text questions, each new piece of information provided by the subject was counted as one detail. A detail was coded as correct if it matched the descriptions in the answer key. For example, as the Black perpetrator was wearing black jeans, both “jeans” and “black” would be counted as correct details. In contrast, if a participant reported that the pants had white stripes, this would be counted as two incorrect details, as the pants were neither white nor did they have stripes. Inter-rater reliability was high for both correct (*r* = .93) and incorrect details (*r* = .85). Following recommendations by Kocab and Sporer (2016), we used the proportion of correct responses (i.e., the number of correct details divided by the total number of details, correct and incorrect) as a measure of memory accuracy.

#### Details on statistical analyses

For non-significant effects, we calculated Bayes factors (Jeffreys, 1935) with default priors (Rouder et al., 2012) to determine the amount of evidence in favor of the null hypothesis. The Bayes factor *BF*_01_ is defined as the ratio of the probability for the observed data to have arisen under the null hypothesis to the probability for them to have arisen under the alternative hypothesis. For example, a value of *BF*_01_ = 5 would indicate that it is 5 times more likely for the observed data to have arisen under the null hypothesis than under the alternative hypothesis. Note that a value of 1 indicates equally strong evidence in favor of the null hypothesis and against it. To communicate the results, we use classifications proposed in the literature (Wagenmakers et al., 2018). Because Bayes factors account for uncertainties in the data, they can be interpreted with confidence for any sample size (cf. Dienes & Mclatchie, 2018).

### Results

#### Total viewing time

**Fig. 2:**
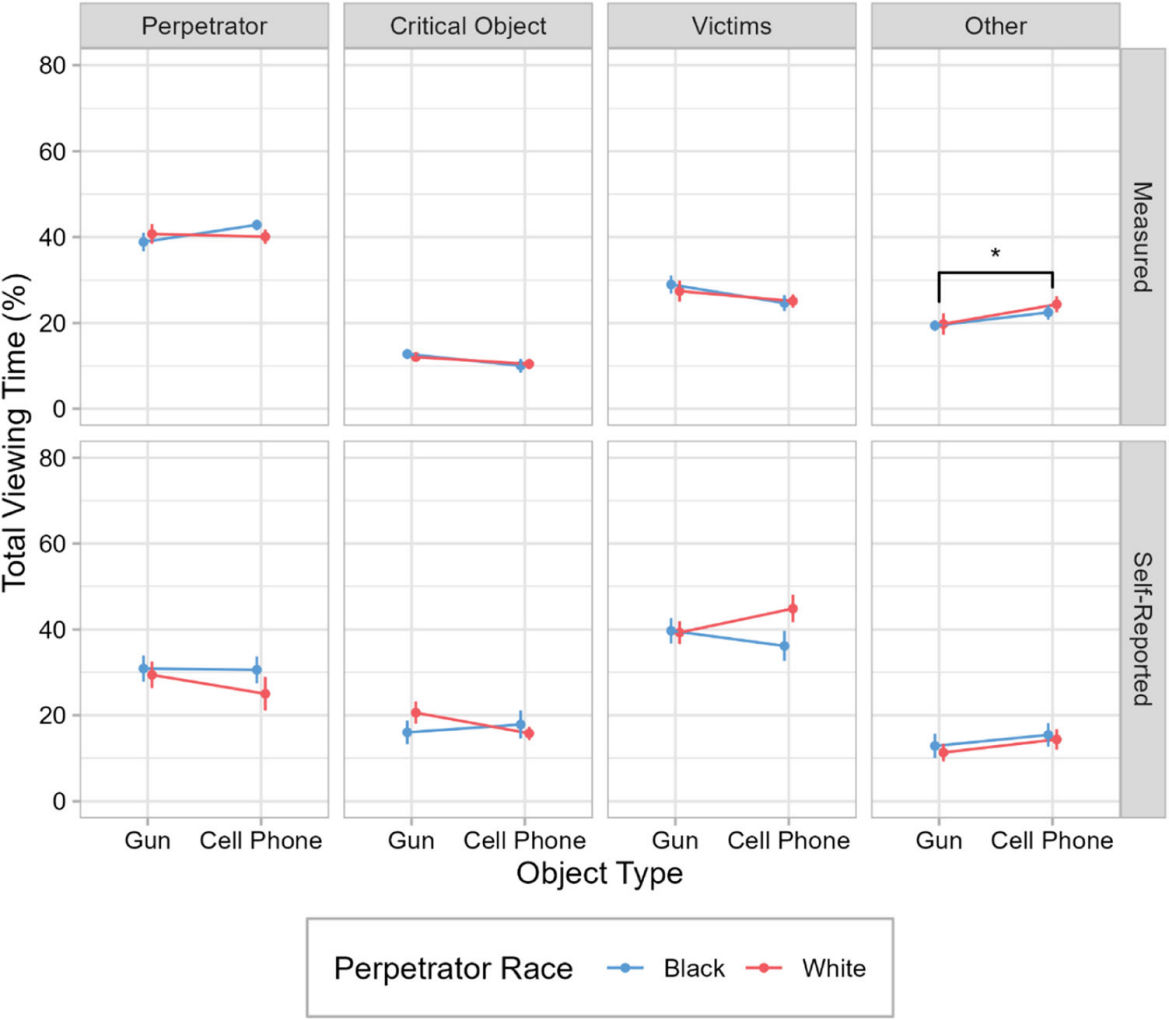
Total viewing times in Experiment 1. Mean total viewing times are shown as a function of object type and perpetrator race. Each column presents the total viewing times for a different region of interest (see panel titles). The rows present objectively measured (top) and self-reported (bottom) data. Error bars are ±1 *SE*. *p < .05. (Color figure online)

The total viewing time (TVT) is the summed duration of all fixations landing within a given ROI. TVT was standardized as the percentage of the duration of the video segments for which eye movement data were analyzed (see above). Table 1 and the top row of Fig. 2 show the relative TVT on the perpetrator, the critical object, the victims, and any area outside of these ROIs as a function of the perpetrator’s race and the object he carried.

**Table 1:** Means and standard deviations for the measured and self-reported relative total viewing times (%) for each region of interest in Experiment 1

Total viewing time	Black perpetrator		White perpetrator	
	Gun	Cell phone	Gun	Cell phone
Measured				
Perpetrator	38.84 (8.98)	42.82 (5.30)	40.71 (9.47)	40.07 (6.90)
Critical object	12.77 (4.70)	10.04 (6.62)	12.06 (4.59)	10.44 (5.05)
Victims	28.99 (8.63)	24.66 (7.75)	27.46 (10.16)	25.12 (6.46)
Other	19.40 (5.20)	22.47 (6.91)	19.78 (10.26)	24.37 (7.63)
Self-reported				
Perpetrator	30.88 (12.53)	30.59 (12.98)	29.41 (12.73)	25.00 (16.20)
Critical object	16.00 (11.32)	17.82 (13.55)	20.59 (10.74)	15.76 (6.32)
Victims	39.71 (12.18)	36.18 (14.42)	39.29 (10.88)	44.88 (13.05)
Other	12.82 (11.80)	15.41 (11.36)	11.29 (8.61)	14.35 (9.76)

Standard deviations are presented in parentheses. *N* = 68 (*n* = 17 for each condition)

TVT was longest for the perpetrator. Unexpectedly, statistical analyses yielded no significant main effect of object type on TVT for the perpetrator, *F*(1, 64) = 0.78, *p* = .382, *BF*_01_ = 2.9 (weak to moderate evidence). The main effect of perpetrator race and the interaction were not significant either (all *p*s ≥ .22, all *BF*s_01_ ≥ 2.1).

Biggs et al. (2013) found a weapon–face trade-off, with longer dwell times (i.e., TVTs) for the weapon being offset by shorter viewing times for the perpetrator’s face. Therefore, we additionally analyzed the allocation of attention to the head of the perpetrator. However, there was no significant main effect of object type for the head ROI either, *F*(1, 64) = 0.01, *p* = .903, *BF*_01_ = 4.0 (moderate evidence). The main effect of perpetrator race and the interaction were also not significant (all *p*s ≥ .66, all *BF*s_01_ ≥ 3.6).

Compared with the TVT for the perpetrator, the TVT for the critical object was much shorter. As predicted, TVT on the gun was numerically longer than TVT on the phone. However, TVTs differed only by approximately 2%, and this difference was not statistically significant, *F*(1, 64) = 2.85, *p* = .096, *BF*_01_ = 1.2 (inconclusive evidence). There was no significant main effect of perpetrator race and no significant interaction of object type and perpetrator race (all *p*s ≥ .66, all *BF*s_01_ ≥ 3.7).

There were no significant effects for TVT on the victims (all *p*s ≥ .10, all *BF*s_01_ ≥ 1.2). For the area outside of the defined ROIs, there was a significant main effect of object type, *F*(1, 64) = 4.19, *p* = .045, η^2^ = .06. Specifically, participants in the gun condition spent less time looking at regions outside of the defined ROIs than participants in the phone condition. The main effect of perpetrator race and the interaction of object type and perpetrator race were not significant (all *p*s ≥ .54, all *BF*s_01_ ≥ 3.4).

#### Self-reported viewing time

The bottom row of Fig. 2 shows the self-reported viewing time as a function of object type and perpetrator race. The most striking difference between the self-reported and the objectively measured TVT is that subjects reported to have looked at the victims the most, followed by the perpetrator, whereas the eye-tracking data revealed the opposite; that is, that the perpetrator attracted the most attention, followed by the victims. Statistical analyses of the self-reported TVTs suggested that there were no significant main effects of object type or perpetrator race and no significant two-way interaction for any of the ROIs (all *p*s ≥ .14, all *BF*s_01_ ≥ 1.5).

#### Time course

Given that we used videos as stimuli, we additionally conducted a time course analysis of the proportion of gaze samples that landed within a given ROI. The eye tracker provided a new gaze sample every millisecond. For the analysis, the raw gaze samples within fixations were binned into 400-ms intervals. Moreover, the number of samples in a given ROI was divided by the total number of valid samples recorded for this interval. Because perpetrator race had no effect on TVT overall, the data were collapsed across race.

**Fig. 3:**
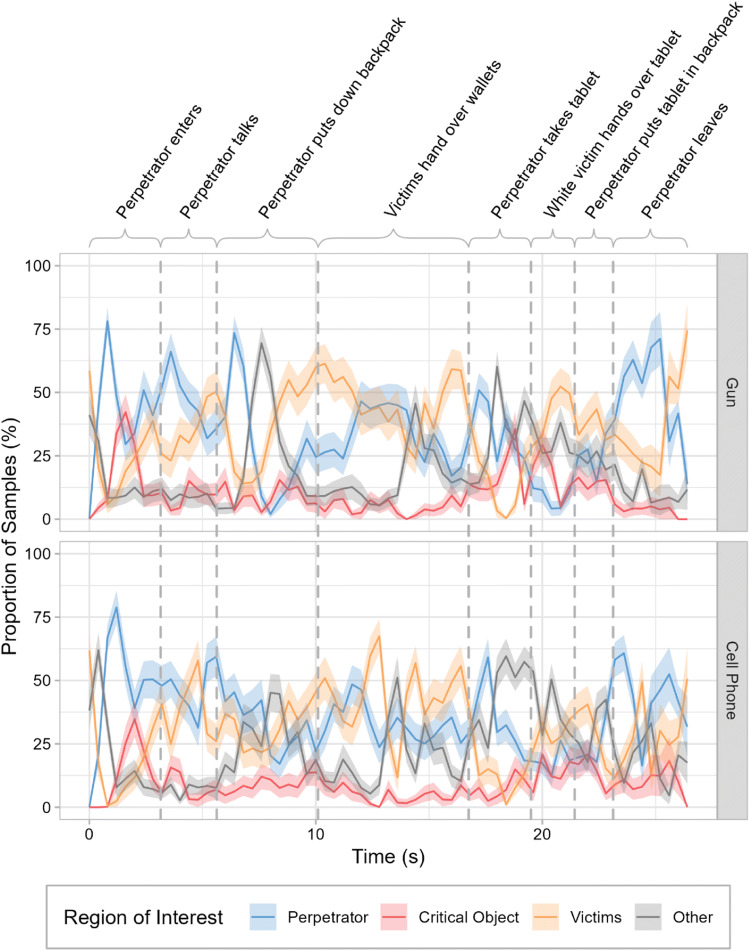
Time course analysis for the eye-movement data in Experiment 1. The proportions of samples that landed within the regions of interest of the perpetrator, the critical object, the victims, and any areas outside of these regions of interest are shown as a function of time for the gun (top) and the cell phone (bottom). Data are shown from the moment the perpetrator enters the room where the two victims are working to the point where he leaves. Vertical lines mark the transitions between actions performed by the people depicted in the videos. Error ribbons are ±1 *SE*. (Color figure online)

Figure 3 shows the proportion of samples that landed within the ROIs of the perpetrator, the critical object, the victims, and any areas outside of these ROIs separately for the gun (top panel) and the phone (bottom panel) conditions. Each graph covers the period from the moment the perpetrator enters the room where the victims are working up to the point where the perpetrator leaves (i.e., we included only the actual robbery, excluding the hallway scene). The data highlight once again that, overall, the critical object received much less attention than the perpetrator or the victims. Both the gun and the phone drew attention primarily at the beginning of the scene as the perpetrator entered the room.

Moreover, the time course analysis provides insights into the coherence of gaze behavior across different observers. For example, observing high values in one ROI and low values in the other two ROIs indicates high inter-observer coherence, in the sense that most participants were focusing on the same region. Conversely, observing similar values for multiple ROIs at a given point in time indicates a lower coherence.

**Fig. 4:**
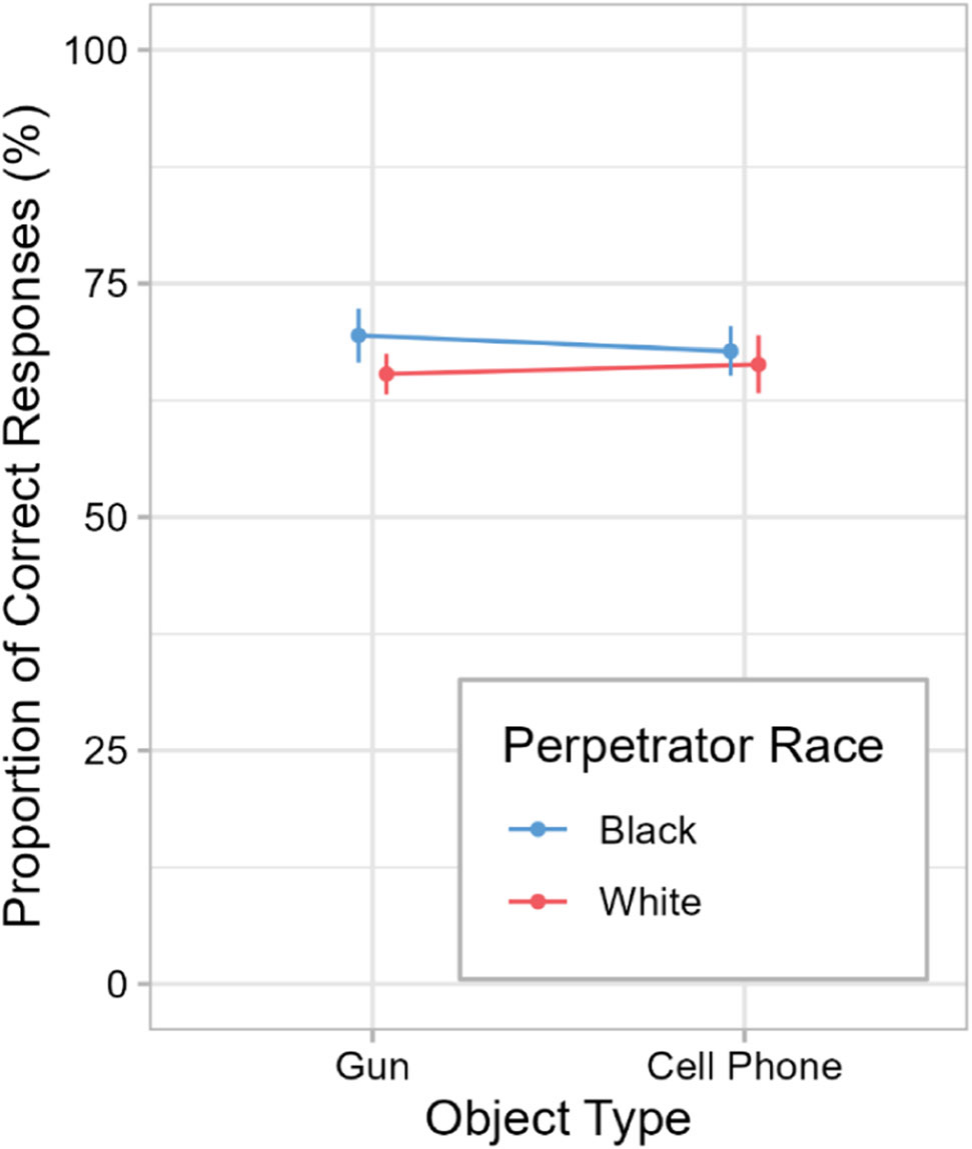
Memory performance in Experiment 1. Mean proportions of correct responses regarding the perpetrator’s appearance in the memory questionnaire are shown as a function of object type and perpetrator race. Error bars are ±1 *SE*. (Color figure online)

For both the gun and the phone, most samples landed within the ROIs of the people involved, i.e., the perpetrator and the victims. However, when the perpetrator was carrying a gun, there was a more pronounced preference for either the perpetrator or the victims for most time points (top panel of Fig. 4). Specifically, between 50 and 75% of the samples landed within one of these ROIs for many bins. In the phone condition, the preference for one of the interacting parties was not as pronounced as in the gun condition.

#### Memory questionnaire

Figure 4 and Table 2 show the proportion of correct responses as a function of object type and perpetrator race. Contrary to expectations, object type had no significant effect on memory accuracy, *F*(1, 64) = 0.01, *p* = .908, *BF*_01_ = 4.0 (moderate evidence). The main effect of perpetrator race and the interaction of object type and perpetrator race were not significant either (all *p*s ≥ .31, all *BF*s_01_ ≥ 2.5). Thus, the hypotheses regarding perpetrator race were also not confirmed. When correct and incorrect details were analyzed separately, there were no significant main effects or interactions either (all *p*s ≥ .24, all *BF*s_01_ ≥ 2.2).

**Table 2:** Means and standard deviations for the memory accuracy (%) and the stereotype ratings in Experiment 1

Measure	Black perpetrator		White perpetrator	
	Gun	Cell phone	Gun	Cell phone
Proportion correct	69.43 (11.79)	67.77 (10.91)	65.34 (9.02)	66.36 (12.67)
Stereotype awareness	7.82 (1.07)	7.29 (1.31)	7.50 (2.68)	7.06 (1.64)
Stereotype endorsement	4.59 (1.23)	4.88 (0.93)	4.94 (1.65)	4.76 (1.44)

Standard deviations are presented in parentheses. *N* = 68 (*n* = 17 for each condition, except for the stereotype ratings in “White Perpetrator – Gun,” for which *n* = 16). Stereotype awareness and stereotype endorsement were rated on a scale from 0 to 10, with 5 indicating no difference between Black and White men

#### Stereotype awareness and endorsement

To reiterate, participants were asked whether a Black or a White man was more likely to commit a crime using a weapon according to (a) existing stereotypes and (b) their personal beliefs. The scale ranged from 0 (*White man extremely more likely*) to 10 (*Black man extremely more likely*), with 5 representing an equal likelihood for Black and White men. Results are presented in Table 2. When asked about stereotypes, participants assessed that Black men were associated more strongly with crimes involving weapons than White men (*M* = 7.42, *SD* = 1.75). This rating differed significantly from the midpoint of the scale, *t*(66) = 11.30, *p* < .001, *d* = 1.38 (one-tailed test). There were no significant main effects of object type or perpetrator race and no interaction (all *p*s ≥ .26, all *BF*s_01_ ≥ 2.2). The rating of the participants’ own personal beliefs was close to the midpoint of the scale (*M* = 4.79, *SD* = 1.31) and did not differ significantly from it, *t*(66) = 1.31, *p* = .902, *BF*_01_ = 16.2 (one-tailed test, strong evidence). Again, there were no main effects or interactions of the independent variables (all *p*s ≥ .47, all *BF*s_01_ ≥ 3.1).

### Discussion

While previous investigations on the memory component of the WFE have repeatedly used dynamic stimuli (e.g., Mitchell et al., 1998; Pickel, 2009; Pickel & Sneyd, 2018), existing eye-tracking studies have used only slide shows (E. F. Loftus et al., 1987) or non-narrative images (Biggs et al., 2013). Therefore, the novel contribution of Experiment 1 was to investigate the WFE with dynamic stimuli and eye tracking.

Our results indicate that the attentional effects of weapons are more complex than current theories and previous results suggested. Specifically, we found no significant effect of weapon presence on viewing time for the perpetrator. Viewing times were numerically longer for the weapon than for the neutral object, but this effect was not statistically significant. Bayes factors indicated that the data provide equally strong evidence in favor and against such an effect. Time course analyses showed that both the weapon and the neutral object were looked at primarily at the beginning of the scene. Thus, weapons do not seem to capture attention over extended periods of time. However, the time course data also showed that participants in the weapon condition focused more consistently on one of the interacting parties (i.e., either the perpetrator or the victims). This higher inter-observer coherence indicates that the presence of a weapon altered participants’ interpretation of the scene, leading them to follow the interaction more closely. This is further supported by the fact that subjects in the weapon condition spent less time looking at regions outside of the defined ROIs. Perpetrator race had no significant effects on gaze behavior. Finally, the eye-tracking results indicate that self-reported viewing times are not a suitable substitute for objectively measured viewing times.

We did not observe reduced memory accuracy in the weapon condition, which means that we could not replicate the classic memory-related WFE (e.g., Kim et al., 2014; Pickel, 2009; Pickel & Sneyd, 2018). Perpetrator race did not significantly affect memory accuracy, which is consistent with our eye-tracking results, but contrasts findings by Pickel and Sneyd (2018).

As these results were unexpected, we conducted two additional experiments. In Experiment 2, we tracked participants’ eye movements while they viewed a sequence of static stimuli resembling those used by E. F. Loftus et al. (1987). In Experiment 3, we closely replicated the methodology of Pickel and Sneyd (2018) by conducting an online experiment with unaltered stimuli.

For these follow-up experiments, we dropped perpetrator race as an independent variable. If perpetrator race has a moderating influence on the WFE, the effect should be greater for the White perpetrator than for the Black perpetrator, due to the higher perceived unusualness of the weapon (Pickel & Sneyd, 2018). To maximize the strength of a potential WFE, we therefore used the stimulus material depicting the White perpetrator only.

## Experiment 2

When using video stimuli in Experiment 1, we found no evidence for weapon presence having a significant effect on viewing time for either the perpetrator or the critical object, which is inconsistent with previous eye-tracking studies on the WFE (Biggs et al., 2013; E. F. Loftus et al., 1987). In a classic study, E. F. Loftus et al. (1987) presented participants with slides, which were shown for 1.5 s each. Using slides or photographs rather than videos facilitates the analysis of eye movements (Biggs et al., 2013). However, in real crime scenarios the protagonists and the objects they hold are likely to move. Recent eye-tracking research has demonstrated that motion in dynamic scenes leads to changes in viewing patterns, the degree of which depends on the viewing task (see Nuthmann & Canas-Bajo, 2022, for a review). Most relevantly, Dorr et al. (2010) found differences in gaze behavior for natural versus stop-motion movies.

To explore whether the stimulus presentation mode affects the attention component of the WFE, we conducted a second experiment in which static stimuli were used. To mimic E. F. Loftus et al. (1987), we converted the videos used in Experiment 1 to slide shows. The critical question was whether the weapon would receive more attention than the neutral object this way and whether such a potential attentional shift would come at a cost of viewing time on the perpetrator.

### Methods

#### Design and participants

Object type (gun vs. cell phone) was manipulated between subjects. Due to the reduced design compared with Experiment 1, we used *t* tests to analyze the data. An a priori power analysis yielded a required sample size of *N* = 56 to detect large effects (*d* ≥ 0.8) with one-tailed *t* tests, a power of at least 1 − β = .90, and a significance level of α = .05. The 56 participants (41 women, 15 men) were between 19 and 50 years old (*M* = 25.5 years, *SD* = 6.9). The procedure was the same as in Experiment 1.

#### Stimuli

Slide shows were created based on the videos depicting the White perpetrator. Given that each slide was presented for 1.5 s, there were 56 images for each slide show, leading to a total duration of 84 s. The video frames used for the slides were manually selected to ensure that (a) all important interactions between the protagonists were included and unambiguously depicted (e.g., the handover of the wallets) and (b) the slides showed the same content in the gun and the phone condition. As in Experiment 1, no soundtrack was presented.

#### Apparatus

The stimuli were displayed on a 24.5 in. monitor with a resolution of 1920 × 1080 pixels and a refresh rate of 144 Hz. An SR Research EyeLink 1000 Plus Tower Mount was used to record participants’ binocular eye movements. As in Experiment 1, the viewing distance was 93 cm.

#### Data analysis

The ROIs for each slide were identical to those of the respective frame of the videos in Experiment 1. To ensure consistency across experiments, the assignment of eye-movement data to ROIs was again based on individual samples. As in Experiment 1, the quality of the eye-tracking data was high (see Appendix 2). Subjects’ responses to the memory questionnaire were again evaluated by two naive raters. Inter-rater reliability was high for both correct (*r* = .95) and incorrect (*r* = .85) details. One-tailed tests were used for viewing time on the perpetrator and the critical object and for memory performance.

### Results

#### Total viewing time

**Fig. 5:**
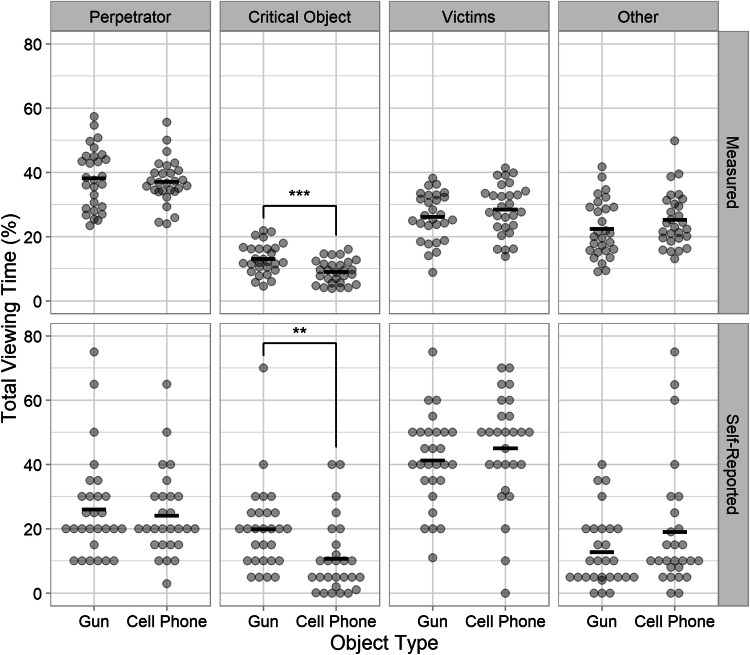
Total viewing times in Experiment 2. The data are shown as a function of object type. Each column presents total viewing times for a different region of interest (see panel titles). The rows present objectively measured (top) and self-reported (bottom) data. Each dot represents an individual participant. Horizontal lines represent the mean. **p < .01. ***p < .001

Table 3 and the top row of Fig. 5 show the TVT on the perpetrator, the critical object, the victims, and any area outside of these ROIs for both the gun and the phone. TVT on the gun was significantly longer than TVT on the phone, *t*(54) = 3.60, *p* < .001, *d* = 0.96, replicating E. F. Loftus et al. (1987). Interestingly, this increase in looking at the gun did not come at the cost of looks to the perpetrator. Instead, there was no significant effect of object type on TVT for the perpetrator, *t*(54) = 0.48, *p* = .684, *BF*_01_ = 5.1 (moderate evidence). When analyzing the TVT on the perpetrator’s head in particular, there was again no significant effect of object type, *t*(54) = 1.34, *p* = .907, *BF*_01_ = 7.8 (moderate evidence). In fact, the mean TVT both for the head ROI and for the entire perpetrator was numerically longer (rather than shorter) in the gun condition compared with the phone condition. There was no significant effect of object type on TVT for the victims or for regions other than the three predefined ROIs (all *p*s ≥ .22, all *BF*s_01_ ≥ 1.9).

**Table 3:** Means and standard deviations for the measured and self-reported relative total viewing times (%) for each region of interest in Experiment 2

Total viewing time	Gun	Cell phone
Measured		
Perpetrator	38.21 (9.53)	37.13 (6.95)
Critical object	13.13 (4.75)	9.06 (3.64)
Victims	26.22 (7.46)	28.51 (7.90)
Other	22.44 (8.88)	25.30 (8.48)
Self-reported		
Perpetrator	26.07 (15.95)	24.19 (13.32)
Critical object	19.82 (13.30)	10.74 (11.53)
Victims	41.29 (14.04)	45.07 (16.98)
Other	12.82 (11.28)	19.07 (19.62)

Standard deviations are presented in parentheses. *N* = 56 (*n* = 28 for each condition, except for the self-reported total viewing times in the phone condition, for which *n* = 27)

#### Self-reported viewing time

As in Experiment 1, subjects wrongly reported to have looked at the victims the most, followed by the perpetrator, whereas the eye-tracking data revealed the opposite (Fig. 5, Table 3). However, in agreement with objectively measured TVTs, participants indicated that they looked longer at the gun than at the phone, *t*(53) = 2.70, *p* = .005, *d* = 0.73 (see also Erickson et al., 2014). Object type did not significantly affect self-reported TVT for any of the other ROIs (all *p*s ≥ .15, all *BF*s_01_ ≥ 1.5).

#### Memory questionnaire

**Fig. 6:**
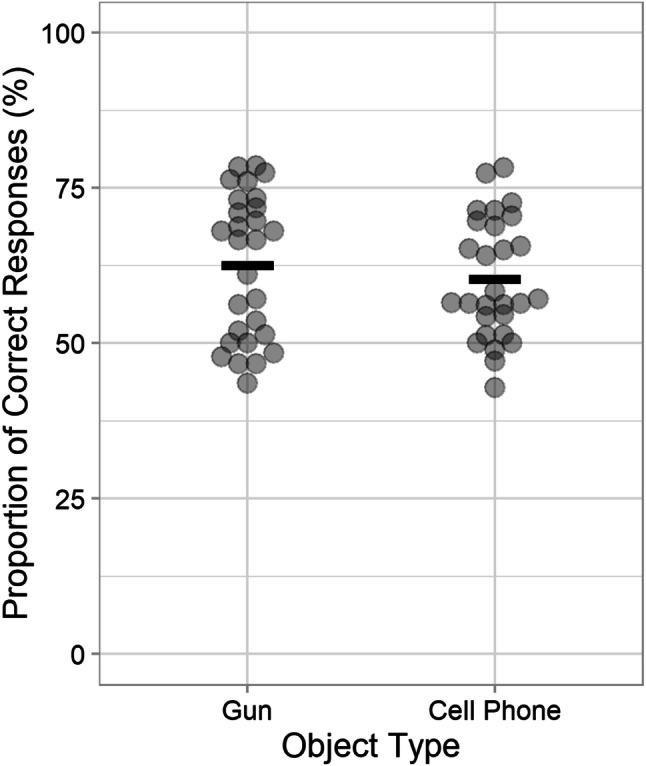
Memory performance in Experiment 2. Each dot presents an individual participant’s proportion of correct responses regarding the perpetrator’s appearance in the memory questionnaire as a function of object type. Horizontal lines represent the mean

Figure 6 shows the proportion of correct responses as a function of object type. The proportion of correct responses did not significantly differ between the gun condition (*M* = 62.44%, *SD* = 11.63) and the phone condition (*M* = 60.26%, *SD* = 9.64), *t*(54) = 0.76, *p* = .776, *BF*_01_ = 5.9 (moderate evidence). When correct and incorrect details were analyzed separately, there were no significant differences either (all *p*s ≥ .39, all *BF*s_01_ ≥ 3.0).

### Discussion

With Experiment 2, we followed up on the results from Experiment 1 by using slide shows instead of video stimuli. We found longer TVTs for the weapon than for the neutral object, which is in agreement with previous eye-tracking studies on the WFE (Biggs et al., 2013; E. F. Loftus et al., 1987). Contrary to predictions by current theories, however, this attention shift did not come at a cost of shorter TVTs on the perpetrator. Our experiment was modelled after E. F. Loftus et al. (1987), who did not report the TVT (or the total number of fixations) for the perpetrator. Using static images but not slide shows, Biggs et al. (2013) reported that their subjects spent less time looking at the faces of people holding weapons relative to neutral objects, a finding which we could not replicate. This difference in results may be due to differences in methodological details between the two studies. Biggs et al. (2013) presented non-narrative images, one by one, for 5 s apiece. Thus, stimulus processing was completely reset at regular intervals. Moreover, there was only one person in each image. In contrast, our consecutive slides told a coherent story, and, when the weapon was visible, both the perpetrator and the two victims were oftentimes competing for the observers’ attention.

Even though object type was found to affect the allocation of attention in some ways, the presence of a weapon did not lead to a reduced ability to recall details about the perpetrator’s appearance. Not finding an effect of object type on memory performance is consistent with Experiment 1, but inconsistent with previously published studies (e.g., Hope & Wright, 2007; Pickel, 1998; Pickel & Sneyd, 2018).

## Experiment 3

We conducted a third experiment to explore whether our failures to replicate the memory-related WFE were due to methodological differences across studies. For the eye-tracking experiments, we made some alterations to the original stimuli used by Pickel and Sneyd (2018). Specifically, we omitted the soundtrack as well as an initial close-up on the critical object. Moreover, the videos were converted to slide shows in Experiment 2.

The goal of our final experiment was to replicate Experiment 1 reported in Pickel and Sneyd (2018) as closely as possible. As in their experiment, the video stimuli included both the close-up on the critical object and the English soundtrack. Our subject recruitment procedures ensured that potential subjects were highly proficient in English. Given that the experiment was conducted in Germany, we were unable to replicate that the first language spoken by the participants matched the language used by the protagonists in the videos. Eye movements were not recorded, and the experiment was run online, which means that we could achieve a larger sample size than in our laboratory eye-tracking experiments. Finding a memory-related WFE in Experiment 3 would suggest that methodological differences were responsible for the absence of the effect in Experiments 1 and 2.

Motivated by the eye-tracking results obtained in Experiment 1, we further extended the original memory test by including questions about the interactions between the perpetrator and the two victims. In Experiment 1, when the weapon was present, observers’ preference for allocating attention to either the perpetrator or the victims changed over time (Fig. 3). Thus, the eye-movement data suggest that there was a particular attentional focus on the interactions among the protagonists, which may enhance observers’ memory for specific actions that were performed. On the other hand, weapons have been shown to impair memory not only for the appearance of the perpetrator, but also for other details of the scene (Kim et al., 2014; Saunders, 2009). Therefore, the analyses of the questions that tested subjects’ memory for the interactions were exploratory in nature.

### Methods

#### Design, participants, and stimuli

Object type (gun vs. cell phone) was manipulated between subjects. Pickel and Sneyd (2018, Experiment 1) tested 266 participants in a study with a 2 × 2 × 2 design. In our Experiment 3, we chose to focus on two of these conditions, yet we tested the same number of participants as Pickel and Sneyd (2018). A sensitivity power analysis indicated that a sample size of *N* = 266 was sufficient to detect small to medium effects (*d* ≥ 0.36) with one-tailed *t* tests, a power of at least 1 − β = .90, and a significance level of α = .05. Participants’ ages ranged from 18 to 84 years (*M* = 30.7 years, *SD* = 13.0); 175 were women, and 91 were men. The original videos from Experiment 1 reported by Pickel and Sneyd (2018) were used as stimuli.

#### Procedure and questionnaire

The procedure was similar to that of the previous experiments, except that participants completed the experiment online with no eye tracking. Subjects could not pause or replay the video. Our additions to the questionnaire were presented only after participants had responded to the original set of questions by Pickel and Sneyd (2018).

As outlined above, we included additional questions regarding the actions performed by the perpetrator and the two victims (e.g., “In total, how many and which objects did the perpetrator take?”). Moreover, we added a number of rating items. Participants were asked to rate how threatening they perceived (a) the perpetrator and (b) the critical object to be. Moreover, subjects were asked to indicate how aroused they were while watching the video. Two more items were added to assess the perceived unusualness of the critical object. Specifically, subjects were asked to indicate how unusual they perceived the object to be (a) in general and (b) in the context of the video. Finally, subjects were asked to rate how well they understood the English dialogue. All items were rated on a scale from 0 to 10, except for the self-reported arousal, which was rated on a scale from 0 to 5.

#### Data analysis

Responses to questions regarding the appearance of the perpetrator as well as details of the actions were evaluated by a second rater only for a subset of 104 participants (cf. Pickel & Sneyd, 2018). Inter-rater reliability was high for both correct (*r* = .98) and incorrect (*r* = .87) details regarding the perpetrator’s appearance and correct (*r* = .97) and incorrect (*r* = .79) details regarding the actions. Only data from the rater who evaluated all participant responses were used for analysis, instead of aggregating across raters as in Experiments 1 and 2. Statistical tests for memory regarding the perpetrator’s appearance were one-tailed, whereas two-tailed tests were used for memory regarding details of the actions. One-tailed tests were used for all rating variables except situational unusualness and linguistic comprehension.

### Results

#### Memory questionnaire

**Fig. 7:**
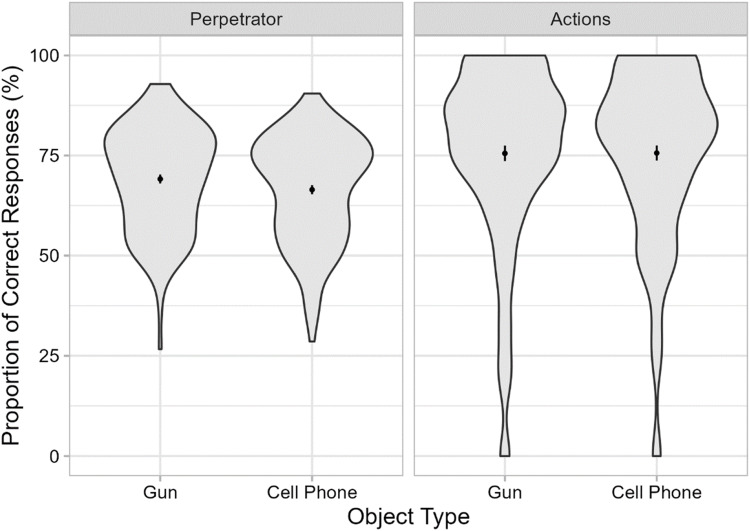
Memory performance in Experiment 3. Mean proportion of correct responses in the memory questionnaire are shown as a function of object type. Data are shown for questions regarding the perpetrator’s appearance (left) and details regarding the actions performed by the depicted people (right). Error bars are ±1 *SE*

Table 4 and Fig. 7 show the effects of object type on memory accuracy for the perpetrator’s appearance and for details of the actions of the people involved. Memory for the perpetrator’s appearance was not significantly worse in the gun condition than in the phone condition, *t*(264) = 1.68, *p* = .953, *BF*_01_ = 19.0 (strong evidence). In fact, memory accuracy was numerically better rather than worse in the gun condition compared with the phone condition. Memory scores for the actions were highly similar in both conditions and did not differ significantly, *t*(264) = 0.03, *p* = .973, *BF*_01_ = 7.4 (moderate evidence). When correct and incorrect details were analyzed separately, no significant differences were found either (all *p*s ≥ .27, all *BF*s_01_ ≥ 4.1).

**Table 4:** Memory accuracy (%) and ratings for threat, unusualness, and linguistic comprehension in Experiment 3

Measure	Gun			Cell phone		
	*n*	*M*	*SD*	*n*	*M*	*SD*
Proportion correct						
Perpetrator	133	69.14	12.84	133	66.47	13.15
Actions	133	75.54	22.37	133	75.63	21.67
Threat						
Object	129	9.21	1.39	127	1.61	2.20
Perpetrator	132	7.10	2.08	133	2.69	1.90
Arousal	133	2.38	1.29	133	1.43	1.19
Unusualness						
General	129	7.29	2.51	127	2.20	3.12
Situational	128	3.48	3.50	126	6.83	3.03
Linguistic comprehension	133	8.00	2.63	133	7.94	2.77

Threat, unusualness, and linguistic comprehension were rated on a scale from 0 to 10, except for arousal, which was rated on a scale from 0 to 5

#### Ratings

Table 4 shows participants’ ratings regarding threat, unusualness, and linguistic comprehension. Overall, the scene was rated as more threatening in the gun condition than in the phone condition (all *p*s < .001).

The two items assessing the perceived unusualness of the critical object showed an interesting dissociation. When asked to assess the object’s unusualness in general, the gun was judged to be more unusual than the phone, *t*(254) = 14.38, *p* < .001, *d* = 1.80. When quizzed about the situational unusualness, however, subjects rated the phone as more unusual than the gun, *t*(252) = 8.15, *p* < .001, *d* = 1.02.

Subjects judged their language comprehension to be high (*M* = 7.97, *SD* = 2.69), with no significant difference between the gun condition and the phone condition, *t*(264) = 0.18, *p* = .856, *BF*_01_ = 7.3 (moderate evidence).

### Discussion

In this experiment, we investigated the effects of weapon presence on memory with the original stimuli used by Pickel and Sneyd (2018) and in a larger sample than in Experiments 1 and 2. As in Experiments 1 and 2, memory for the perpetrator’s appearance was not impaired when he was holding a weapon during the robbery depicted in the videos. Thus, we again failed to find a memory-related WFE.

Since Experiment 3 was designed to be a close replication of Pickel and Sneyd (2018), the question arises why we could not replicate their results. In our experiment, participants’ first language was German and thus did not match the language used by the protagonists in the videos. However, subjects’ self-reported language comprehension was high. One major difference between the study by Pickel and Sneyd (2018) and ours is the cultural background of the participants. Like most studies on the WFE, Pickel and Sneyd (2018) conducted their experiment in the U.S., whereas our data were collected in Germany. It is possible that German participants process gun-related events differently, for example due to the lower rate of homicides involving firearms (Grinshteyn & Hemenway, 2016). However, the diverging results could also be due to differences in other factors such as the heterogeneity of the sample or participant compliance. In online studies in particular, it can be difficult to obtain a balanced sample, control for potential biases, and ensure compliance. The present data alone are not sufficient to distinguish between these explanations. Our data do, however, convincingly show that the presence of a weapon does not always lead to an impairment in memory.

## General discussion

To explain impaired memory for a target person holding a weapon, researchers have suggested that observers shift their attention away from the target and towards the weapon (e.g., E. F. Loftus et al., 1987; Pickel, 2015). Putting this key assumption to a test, we used eye tracking to assess where and for how long observers allocate their attention when viewing a robbery scene. Our Experiment 1 is the first to systematically investigate the attentional effects of weapons using video stimuli. In Experiment 2, we converted the videos to slide-shows of static images (cf. E. F. Loftus et al., 1987) to explore how well attentional effects generalize across different stimulus presentation modes. Experiment 3 was an online study focusing on the memory-related WFE. All stimuli were based on videos for which large effects on memory have previously been found (Pickel & Sneyd, 2018, Experiment 1). Our detailed results offer novel and surprising insights that cast doubt on the hypothesis that weapons draw attention away from the perpetrator for extended periods of time.

For dynamic scenes (Experiment 1), the attentional effects of weapons were different and more complex than previously assumed. If the allocation of attention towards the critical object (i.e., the gun or the phone) is an important condition for the WFE to emerge, we would expect observers to look at the critical object fairly extensively. However, both the weapon and the neutral object attracted only 10%–15% of observers’ viewing time, which means that they were looked at for much shorter durations than the depicted people. In total, the weapon attracted slightly more gaze than the neutral object, but this difference was not significant at an alpha of 5%. The corresponding Bayes factor indicated that our data provide as much evidence in favor of such an effect as they do against it. Viewing time on the perpetrator was not significantly affected by weapon presence. Thus, we found no evidence in favor of the key assumption made by current theories that a weapon draws attention away from the perpetrator.

A novel time course analysis additionally revealed how the allocation of attention changed over time (see Fig. 3). Both critical objects were looked at primarily at the beginning of the scene and only infrequently thereafter. Participants in the weapon condition focused their attention more consistently on either the perpetrator or the victims for most points in time. Weapon presence also led participants to spend less time looking at regions outside of the defined ROIs (i.e., the perpetrator, the critical object, and the victims). These findings indicate that participants were more involved in following the interaction of the perpetrator and the victims when the former was carrying a weapon compared with a neutral object. Scrivner et al. (2019) similarly found that violent as opposed to non-violent scenes led participants to focus more strongly on the interaction of the depicted people, with their gaze shifting towards points of contact rather than objects held by the interacting people (e.g., weapons).

To test whether the unexpected results of Experiment 1 were due to using dynamic stimuli, we turned the video material into slide shows (Experiment 2), mimicking the methodology of E. F. Loftus et al. (1987). Like E. F. Loftus et al. (1987), but in contrast to Experiment 1, we found significantly longer viewing times for the weapon than for the neutral object. Thus, the attention capturing effects of weapons may be more pronounced for (series of) static images than for dynamic stimuli. This difference in results highlights the importance of studying attentional phenomena under naturalistic circumstances. Finding differences in gaze behavior for videos and slide shows is generally consistent with results by Dorr et al. (2010), who showed that the attentional processing of natural dynamic stimuli differs from that of series of static images and heavily edited movie trailers.

Note that in other important aspects, slide shows (Experiment 2) and videos (Experiment 1) yielded similar results. In both experiments, the weapon attracted little attention overall. Moreover, viewing time on both the perpetrator as a whole and his face in particular (cf. Biggs et al., 2013) was not significantly reduced by the presence of a weapon in either experiment. E. F. Loftus et al. (1987) did not report viewing time on the perpetrator, and the divergence with findings reported by Biggs et al. (2013) is likely due to methodological differences (see the discussion of Experiment 2).

Erickson et al. (2014) measured the allocation of attention subjectively via self-report rather than objectively via eye tracking. Therefore, a secondary objective of our study was to assess the level of agreement between the two methods. Participants correctly assessed that they allocated most of their viewing time to the depicted people (rather than the critical object or other aspects of the scene). However, subjects indicated to have spent most of their looking time on the victims, when in fact they paid most attention to the perpetrator. Moreover, between-subject variability was considerably higher for self-reported than for objectively measured data (see Fig. 5).

In their study, Erickson et al. (2014) used interactive slide shows. With this material, they found a self-reported attentional shift towards a weapon. Similarly, when using slide shows in Experiment 2, we found that participants reported to have spent more time looking at the weapon than at the neutral object. Given that this self-reported attentional shift matches a shift in objectively measured viewing time, an attentional shift towards weapons may indeed be consciously accessible, at least for slide-show stimuli.

When considering the data for videos and slide shows together, we found that, overall, self-report measures were not very accurate representations of actual gaze behavior. These results complement previous findings suggesting that observers have only limited insight into their own eye movements (e.g., Clarke et al., 2017; Foulsham & Kingstone, 2013; Võ et al., 2016). In any case, more elaborate investigations of attentional allocation (e.g., the time course analyses presented in Fig. 3) will always require eye-tracking data.

According to the attention-shift hypothesis, an attentional shift away from the perpetrator towards the weapon is responsible for impairing observers’ ability to recall details about the person holding the weapon. In both eye-tracking experiments, the presence of a weapon did not reduce the viewing time for the perpetrator. Therefore, not finding a memory-related WFE in these experiments is consistent with patterns of attention allocation observed in the data. Not finding the predicted memory effect is, however, inconsistent with results by Pickel and Sneyd (2018), who used similar stimulus material and reported large memory effects. Therefore, in Experiment 3, we presented the unaltered videos of Pickel and Sneyd (2018) online and replicated their sample size. Again, no memory-related WFE emerged. Bayes factors indicated that the data from all our experiments provide evidence against a memory-related WFE, with evidence being moderate for Experiments 1 and 2 and strong for Experiment 3. Moreover, several recent studies similarly found weapon presence to have no effect on or even enhance memory for at least some measures or experimental conditions (Harvey et al., 2020; Harvey & Sekulla, 2021; Mansour et al., 2019; Nyman et al., 2020).

The fact that our results diverge from findings by Pickel and Sneyd (2018) demonstrates that, even when employing very similar stimuli and methodology, the memory-related effects of weapons can differ drastically. It remains unclear whether cultural differences between German and U.S.-American participants or other differences between their sample and ours are responsible for these opposing findings. Future research is needed to identify the specific sample characteristics moderating the memory-related WFE.

Results from Experiment 2 show that an attentional focus on a weapon can be observed in a sample that does not show a memory effect. Conversely, other studies on eyewitness testimony have shown that memory effects are not always caused by attentional phenomena (Cahill et al., 2003; Echterhoff & Wolf, 2012). Therefore, future research linking the memory-related WFE to attentional processes should directly and objectively measure the allocation of attention instead of inferring an attentional effect based on differences in memory accuracy. Moreover, future studies on the attentional component of the WFE should strive to use dynamic scene stimuli, as has been done in numerous studies on the memory-related WFE (e.g., Mitchell et al., 1998; Pickel, 2009; Pickel & Sneyd, 2018).

All current experiments were based on one scene only. This is typical for studies on the WFE (e.g., Carlson et al., 2017; E. F. Loftus et al., 1987; Pickel & Sneyd, 2018), because after participants responded to questions about one particular crime scene, their viewing strategies and responses may change when shown additional similar stimuli. Strictly speaking, the inferences that can be drawn from each of these studies apply only to the particular stimulus used (see Yarkoni, 2022). Note that we adapted our stimulus material from a study in which large effects on memory were observed (Pickel & Sneyd, 2018). Therefore, the absence of a memory-related WFE in our experiments is unlikely to be due to specific stimulus properties. However, future research should explore the degree to which attentional effects in particular generalize across populations of stimuli.

One stimulus characteristic potentially affecting the WFE may be the depiction of victims. The material used in the present study showed two men being robbed. This led to the perpetrator and the victims oftentimes competing for the observers’ attention, with the victims being the second-most looked at region after the perpetrator for both the videos in Experiment 1 and the slide shows in Experiment 2. Interestingly, participants subjectively reported to have paid more attention to the victims than the perpetrator. The WFE has been found both for stimuli including victims (e.g., Kim et al., 2014; E. F. Loftus et al., 1987; Pickel, 1998) and for scenes without visible victims (e.g., Biggs et al., 2013; Carlson et al., 2016; Kramer et al., 1990). However, the depiction of victims has not yet been manipulated experimentally in the context of the WFE. It is possible that the presence vs. absence of victims as well as their characteristics (e.g., gender, race) and demeanor (e.g., emotional vs. calm) modulate the effects weapons have on attention or memory.

Finally, we compared a weapon to a neutral control object only. Non-threatening objects that do not fit into the context of the scene (i.e., unusual objects) have been shown to impair memory in a similar manner as weapons do (e.g., Mitchell et al., 1998; Pickel, 1998). However, the attentional effects of weapons and unusual objects have not yet been compared directly via eye tracking. Such a comparison could help to further evaluate whether unusualness is indeed the key factor responsible for the WFE.

### Conclusion

The main results of our study are that weapons (a) do not capture attention over extended periods of time and (b) do not necessarily draw gaze away from the perpetrator. Instead of a simple shift from the perpetrator towards the weapon, the attentional effects of weapons seem to be more complex than current theories suggest. More generally, our findings indicate that it is important to study attentional allocation under naturalistic viewing conditions. This is especially true for effects with strong practical implications, such as the WFE.

## Appendix 1

### Software details

Power analyses were conducted using G*Power (Version 3.1.9.7; Faul et al., 2007). The eye-tracking experiments were implemented in MATLAB (Version R2019b) using the Psychophysics Toolbox extension (Version 3.0.16; Brainard, 1997; Kleiner et al., 2007; Pelli, 1997), which incorporates the EyeLink Toolbox (Cornelissen et al., 2002). The online experiment was implemented in LimeSurvey (Version 3.23.3). We used the SR Research Data Viewer software (Version 4.2.1) to process the raw eye-movement data and to create elliptical ROIs for the critical objects and the head of the perpetrator. The ROIs comprising the people depicted in the stimuli were created with Python (Version 3.7.9) using the libraries *NumPy* (Version 1.21.6; Harris et al., 2020), *OpenCV* (Version 4.6.0.66; Bradski, 2000), *pandas* (Version 1.3.5; McKinney, 2010), and *PixelLib* (Version 0.7.1; Olafenwa, 2021). The eye-movement data and the behavioral data were analyzed with R (Version 4.2.1) using the packages *BayesFactor* (Version 0.9.12-4.4; Morey & Rouder, 2022), *effsize* (Version 0.8.1; Torchiano, 2020), *ez* (Version 4.4-0; Lawrence, 2016), *ggplot2* (Version 3.3.6; Wickham, 2016), *ggsignif* (Version 0.6.3; Ahlmann-Eltze & Patil, 2021), *gtable* (Version 0.3.1; Wickham & Pedersen, 2022), *huxtable* (Version 5.5.0; Hugh-Jones, 2022), *knitr* (Version 1.40; Xie, 2015), *pBrackets* (Version 1.0.1; Schulz, 2021), *scales* (Version 1.2.1; Wickham & Seidel, 2022), and *tidyr* (Version 1.2.1; Wickham & Girlich, 2022).

## Appendix 2

### Quality assessment of the eye-tracking data

Following guidelines by Holmqvist et al. (2022), we empirically assessed the quality of the eye-tracking data in Experiments 1 and 2. Specifically, we determined the accuracy as the average validation error, the precision as the root-mean-square sample-to-sample deviation, and data loss as the proportion of missing samples. Appendix Table 5 shows that, for both experiments, accuracy and precision were good, and data loss was low.

**Table 5:** Means and standard deviations for data quality criteria for the eye-tracking data in Experiments 1 and 2

Experiment	Average validation error	RMS-S2S	Missing samples
Experiment 1	0.32 (0.08)	0.0222 (0.0050)	1.49 (1.76)
Experiment 2	0.30 (0.07)	0.0162 (0.0039)	1.44 (1.82)

Standard deviations are presented in parentheses. RMS-S2S = root-mean-square sample-to-sample deviation. Average validation error and RMS-S2S are in degrees of visual angle; missing samples are in percentages
